# Increased intra-individual variability among individuals with ADHD: first evidence from numerosity judgment and verbal and quantitative reasoning

**DOI:** 10.1017/S0033291724001892

**Published:** 2024-10

**Authors:** Meir Barneron, Noa Saka, Shaul Shlepack, Aseel Khattab, Yehuda Pollak

**Affiliations:** 1 The Seymour School of Education, Hebrew University of Jerusalem, Jerusalem, Israel; 2 National Institute for Testing and Evaluation, Jerusalem, Israel

**Keywords:** ADHD, Increased Intra-Individual Variability, Numerosity Judgments, Verbal and Quantitative Reasoning

## Abstract

**Background:**

This article presents the results of two studies investigating increased intra-individual variability (IIV) in the performance of individuals with attention deficit hyperactivity disorder (ADHD), in two cognitive domains: numerosity judgments and quantitative and verbal reasoning.

**Methods:**

Study 1, a pre-registered experiment, involved approximately 200 participants (42.66% female; mean age: 36.86; standard deviation of age: 10.70) making numerical judgments at two time-points. ADHD-symptom severity was assessed on a continuous scale. In Study 2, we collected the data of approximately 3000 examinees who had taken a high-stakes admissions test for higher education (assessing quantitative and verbal reasoning). The sample comprised only people formally diagnosed with ADHD. The control group consisted of approximately 200 000 examinees, none of whom presented with ADHD.

**Results:**

The results of Study 1 revealed a positive correlation between IIV (distance between judgments at the two time-points) and ADHD symptom severity. The results of Study 2 demonstrated that IIV (distance between the scores on two test chapters assessing the same type of reasoning) was greater among examinees diagnosed with ADHD. In both studies, the findings persisted even after controlling for performance level.

**Conclusions:**

The results indicate that individuals with ADHD, *v.* those without, exhibit less consistent numerosity judgments and greater fluctuation in performance on verbal and quantitative reasoning. The measurement of the same psychological constructs appears to be less precise among individuals with ADHD compared to those without. We discuss the theoretical contributions and practical implications of our results for two fields: judgment and decision-making, and assessment.

Attention deficit hyperactivity disorder (ADHD) is a highly prevalent neurodevelopmental condition defined by persistent symptoms of inattention and/or hyperactivity–impulsivity interfering with functioning or development (APA, 2013). A hallmark feature of ADHD is increased intra-individual variability (IIV) (Kuntsi & Klein, 2011; Tamm et al., 2012), meaning fluctuations in behavioral and cognitive functioning. IIV has been operationalized as short-term changes in performance on a single measure across time. This index reflects transient changes in behavior that can be distinguished from more enduring changes, such as learning and development (MacDonald, Nyberg, & Bäckman, 2006).

In day-to-day life, increased IIV in individuals with ADHD has been demonstrated in the symptoms that they display, in relation to inattention, hyperactivity, and impulsivity (Schmid, Stadler, Dirk, Fiege, & Gawrilow, 2020), classroom behavior (Rapport, Kofler, Alderson, Timko, & DuPaul, 2009), and mood (Skirrow, McLoughlin, Kuntsi, & Asherson 2009), among others. More often, ADHD-related IIV is observed in studies examining cognitive tasks, processing speed, sustained attention, response inhibition, and working memory (for a review, see Kuntsi and Klein, 2011).

The vast majority of these studies operationalize IIV as the variability around the mean reaction time over time (Kofler et al., 2013). Notably, response-time variability is found to relate more often to inattention symptoms but also to hyperactivity/impulsivity symptoms (Tamm et al., 2012), and one study reported significant relationships between response-time variability and each of the 18 ADHD symptoms established by the American Psychiatric Association (APA., 2013; Epstein et al., 2003). Importantly, only rarely has IIV in performance quality or accuracy been examined. The few studies that report increased IIV in terms of performance quality are based on working memory tasks (Friedman, Rapport, & Fabrikant-Abzug, 2022) and time estimation (Bluschke, Zink, Mückschel, Roessner, & Beste, 2021; Pollak, Kroyzer, Yakir, & Friedler, 2009; Toplak, Rucklidge, Hetherington, John, & Tannock, 2003). More recently, studies have shown that individuals with high ADHD symptoms exhibited increased variability in both their willingness to take risks and their willingness to tolerate delays (Gabrieli-Seri, Ert, & Pollak, 2022; Pollak, Gabrieli Seri, & Dekkers, 2020).

A key characteristic of most of these studies is that they focus on participants' performance on neuropsychological tasks, in which IIV was primarily operationalized as response-time variability on multiple trials across a few seconds. It is important, however, to broaden the study of ADHD-related IIV to other cognitive domains and timeframes (e.g. minutes and hours), especially those that are crucial for success in many commonplace settings, including work and academic performance. The lack of research concerning ADHD-related IIV in those domains, and across longer timelines, is unfortunate, considering the significant functional impairment individuals with ADHD face in everyday life and academic tasks (APA, 2013).

## The present studies

We conducted two studies to address the abovementioned gaps in the literature by investigating ADHD-related IIV in numerosity judgments, and in performance in verbal and quantitative reasoning across a minutes-to-hours timeframe.

On the one hand, in numerosity-judgment tasks, people are asked to make numerical estimations of quantities relating to scenarios that frequently arise in real-life situations. These might include, for instance, estimations of the distance separating two vehicles, the number of calories in different foods, the years when historical events occurred, or the number of guests at a party.

On the other hand, verbal and quantitative reasoning both require individuals to apply knowledge and logical reasoning to solve problems, and to analyze and interpret information presented in various formats. These formats variously necessitate proficiency in language comprehension, logical inference, critical thinking skills, the use of numbers and mathematical concepts, and mathematical problem-solving. These abilities are crucial in daily work and academic functioning because the ability to reason is of central importance for thinking about the causes of events, evaluating the correctness of assumptions and validity of arguments, or developing new ideas (Bruine de Bruin & Slovic, 2021; Wilhelm, 2005).

## Controlling for alternative explanations

Numerous large-scale studies and meta-analyses have linked ADHD to lower performance in tests of attention, memory, executive functioning, and decision-making (for reviews, see Faraone et al., 2021; and Pievsky and McGrath, 2018). Similar findings have been reported with respect to academic achievements and skills (see Baweja, Mattison, and Waxmonsky, 2015, for a review).

In theory, the relationship between ADHD symptoms and IIV could be confounded by the accuracy (or otherwise) of the participants' performance. People making more accurate judgments are typically expected to exhibit lower IIV, while those making judgments that are less accurate than average might exhibit higher IIV, irrespective of their ADHD symptoms. If we take the case, for example, of a person who gives the right answer first time around, we might reasonably assume that this implies they *know* the correct answer and are therefore likely to get it right on a different occasion too. By contrast, if someone gives the wrong answer the first time around, we might assume that this is because they do *not* know the correct answer and that they might well provide a different answer on another occasion (which may also happen to be correct).

Consider, for example, a person making an accurate numerical judgment. It is logical to presume that this person will make similarly accurate judgments on different occasions, reducing the variability over time. In contrast, a person making a less accurate judgment might logically be expected to exhibit higher variability. Therefore, theoretically, the finding that individuals with ADHD exhibit higher IIV might be explained by the fact that they are also less accurate in their judgments. This confounding factor should be carefully considered and controlled for, to ensure clarity around the link between ADHD symptoms and IIV. For this reason, when exploring the relationship between ADHD and increased IIV, our studies controlled for participant accuracy. This method enabled us to investigate the claim that, given two individuals with similar levels of performance, one with ADHD and the other without, the former will exhibit greater fluctuation in performance across time.

## Research transparency statement

Study 1 was pre-registered at https://aspredicted.org/5NG_FB2. The data, the code, and the materials for the study can be obtained on demand from the corresponding author. The authors declare no conflict of interest.

## Study 1: numerosity judgments

In this study, participants were asked to make quantitative estimates of the number of candies in a series of jars, at two separate points in time. IIV was defined as the absolute difference between the estimates made on these two occasions. ADHD symptoms were also assessed. We hypothesized that ADHD symptoms would be positively correlated with IIV. Based on the existing literature, we also anticipated that stronger ADHD symptoms would be associated with less accurate estimates. To rule out the possibility that the relationship between IIV and ADHD could be attributed to a difference in level of performance, Study 1 controlled for participants' accuracy.

### Method

#### Participants

We recruited 218 individuals via the MTurk platform. Participants were not recruited according to any particular characteristic. A power calculation confirmed that this sample size was sufficient for detecting a small-to-medium effect size of *r* = 0.19 with a 95% confidence level and a power of 0.80. The participants took part in an 11-minute study and received $2 in compensation for their contribution. The mean age of the sample was 36.86, the standard deviation was 10.70, and 42.66% of our participants were females.

#### Materials and procedure

To familiarize the participants with the research materials (as is common practice in judgmental tasks, e.g. Yaniv and Choshen-Hillel, 2012), they were first presented with two photographs, each showing a jar containing candies, with the correct number of candies in the jar displayed alongside it.

The experimental procedure involved three main phases. Phase 1 consisted of exposing the participants to nine photographs, each featuring a jar containing candies that had not been presented before. All the participants were shown the same nine photographs, but the sequence in which the jars were shown was randomized. In each trial, participants were asked to estimate the number of candies in the jar and were given a time limit of 30 s to make their estimates. The actual number of candies in the jars ranged from 120 to 607. Participants were informed that the person who achieved the most accurate estimates would receive an additional payment of $1. Participants were asked to keep their estimates within the range of 90–750.

The instructions for Phase 2 were displayed immediately after Phase 1 ended. This second phase consisted of presenting the same 9 jars (again, randomly sequenced for each participant), for the same duration as in Phase 1. In this phase, participants were asked to provide their *current* best estimates and were told that these could be different from the answers they provided in Phase 1. As before, they were informed that the participant who made the most accurate estimates in this phase would receive an additional payment of $1. Therefore, participants in both Phase 1 and Phase 2 were motivated to deliver their best and most current estimates.

In Phase 3, participants were asked to fill out the Adult ADHD Self Report Scale (ASRS-V1.1; Kessler et al., 2005), a questionnaire measuring ADHD symptoms, adapted for computer presentation, allowing for the continuous scaling of ADHD symptoms. For this study, a dimensional model of ADHD was adopted, based on taxometric and genetic evidence that supports the use of a dimensional conceptualization of ADHD for research purposes (Coghill & Sonuga-Barke, 2012). The ASRS-V1.1 consists of 18 items, each corresponding to the DSM-IV diagnostic criteria for ADHD. Participants rated the frequency of occurrence of each symptom (item) on a 5-point Likert scale, ranging from ‘never’ to ‘very often’. The questionnaire authors report high validity and internal consistency (a Cronbach's *α* of 0.88 in assessing ADHD in adults). The questionnaire's reported sensitivity is 68.4% and its specificity is 99.6% (Adler et al., 2006). After answering the questionnaire, participants indicated their age and gender. They were also asked whether they had previously been diagnosed with ADHD. Among our participants, 30.28% reported having a previous ADHD diagnosis. Note that, although participants reported whether or not they had previously received an ADHD *diagnosis*, in the present study, our main hypothesis is about the relationship between IIV and ADHD *symptoms* as measured by the ASRS-V1.1. The ASRS-V1.1 provides a continuous measure of ADHD symptoms (as opposed to delivering a binary diagnosis).

### Results

Initially, we recruited 255 participants. The pre-registration involved exclusion rules to ensure the quality of the data. Regarding Phases 1 and 2, the rules required participants to provide estimates in the range of 90–750 and to do so for at least seven (out of nine) jars. For Phase 3, exclusion rules required participants to answer at least 80% of the ADHD questionnaire. In addition, in one item, participants were told ‘For this specific item, please do not select any option’. Participants who chose one option were excluded. Following those rules, 37 participants (14.51% of the initial sample) were excluded. Therefore, the final sample consisted of 218 participants.

From Phases 1–3, we derived three variables. The first variable was the IIV, which served as our sole dependent variable. To calculate IIV for each participant and jar, we computed the absolute distance between the estimates made in Phase 1 *v.* 2. These absolute distances were then standardized by jar, to account for the variance in the answers to the different jars. Next, we calculated the IIV for each participant by computing the mean of the standardized absolute distances across the jars. Finally, IIVs were standardized across participants, to obtain a dependent variable with an intuitive meaning.

The second variable was the Absolute Error (AE), which represented the overall accuracy of a participant's judgment, across jars and phases. To calculate the AE for each participant and jar in Phases 1 and 2 separately, we computed the absolute difference between their estimate and the correct number of candies. These AEs were then standardized by jar, for Phases 1 and 2 separately. Next, we computed the mean AEs for each participant in Phases 1 and 2 separately, by averaging the standardized AEs across jars. As a result, each participant had a mean AE score for Phase 1 and for Phase 2. Then we averaged the two mean AEs for each participant. Finally, the AEs were standardized across participants. Note that higher AEs indicate lower-performing participants.

The third variable was the assessment of ADHD symptoms. This measure represented the extent to which a participant experienced ADHD symptoms in their day-to-day life. An ADHD score was calculated for each participant by averaging the scores of all 18 items. ADHD scores were then standardized across participants. Higher values indicated participants with stronger ADHD symptoms.

Therefore, each participant in the dataset was characterized by three variables: IIV, AE, and the ADHD score. In accordance with our pre-registration, we analyzed the data using two main linear regression models, one predicting IIV from ADHD scores, and a second predicting IIV from ADHD scores and AEs, therefore controlling the relationship between IIV and ADHD for participants' accuracy. Note that, as our variables were standardized, the coefficients obtained from our models can be interpreted in terms of standardized (beta) coefficients.

**Figure 1.**
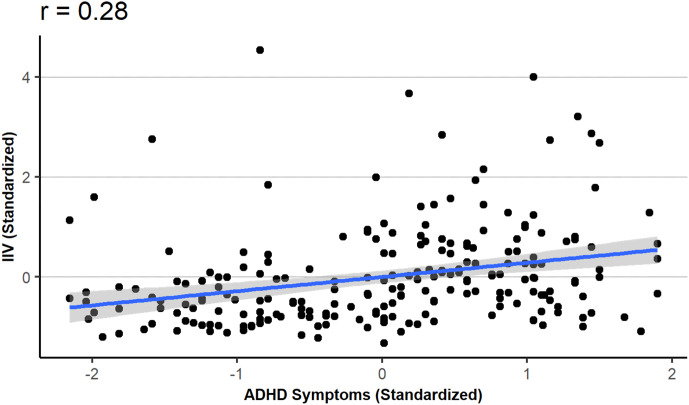
Intra-individual variability (IIV) as a function of ADHD symptoms.

**Figure 2.**
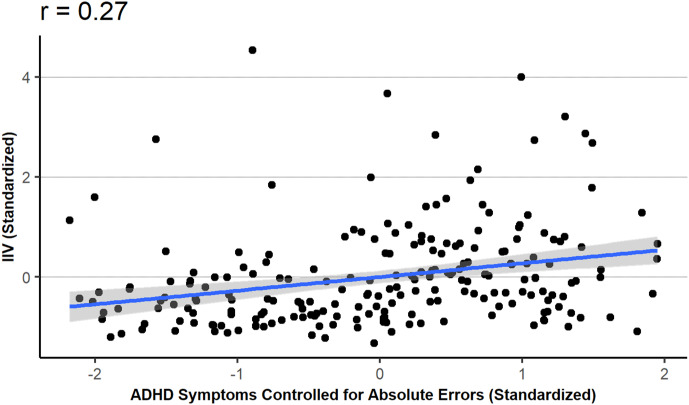
Intra-individual variability (IIV) as a function of ADHD symptom while controlling for absolute errors. *Note*: the x-axis represents the (standardized) residual from the linear model predicting ADHD symptoms from absolute errors.

Figures 1 and 2 display the main results of Study 1. Figure 1 displays the correlation between ADHD symptoms and IIV; Figure 2 displays the correlation between ADHD symptoms and IIV while controlling for participants’ accuracy.

First, we investigated the relationship between IIV and ADHD. We did this by conducting a linear regression analysis predicting IIVs from ADHD scores. The results revealed that higher ADHD scores were associated with higher IIVs (*b* = 0.28, *t*(216) = 4.36, *p* < 0.001). We can therefore affirm that a moderate association was found between ADHD symptoms and IIV (*r* = .28; see Fig. 1).

Second, we investigated the relationship between ADHD and AEs. The linear model, predicting AEs from ADHD symptoms, revealed a positive association between the two, indicating that higher ADHD scores were associated with decreased accuracy (*b* = 0.21, *t*(216) = 3.16, *p* = 0.002). The correlation between the AEs and ADHD was 0.21.

Our third, and main, analysis investigated the relationship between IIV and ADHD while controlling for the AE (overall accuracy). The linear model predicted IIV from both ADHD scores and AEs. Results indicated that higher ADHD scores were associated with higher IIVs (*b* = 0.27, *t*(215) = 4.27, *p* < 0.001). Higher AEs were also associated with higher IIVs (*b* = 0.20, *t*(215) = 3.05, *p* = 0.003). The semi-partial correlation between IIV and ADHD symptoms, controlling for AE, was 0.27 (see Fig. 2).

The above analyses found evidence for the hypothesis that higher ADHD symptoms are associated with greater IIV and that this relationship holds even when controlling for participants’ accuracy.

#### Exploratory analyses

Next, we conducted a series of exploratory analyses to gain further insight into our data and test the robustness of our findings. These analyses were not pre-registered.

In the first exploratory analysis, we examined the potential influence of demographic variables on our results. The linear model predicted IIV from ADHD scores, AEs, age, and gender. In this analysis, we excluded one participant who marked ‘other’ in response to the gender question, as we were unable to assess the impact of one category including only a single instance. The results indicated a positive association between IIV and ADHD symptoms (*b* = 0.28, *t*(212) = 4.27, *p* < 0.001) and a positive association between IIV and AEs (*b* = 0.20, *t*(212) = 3.07, *p* = 0.002). However, we did not observe any effect exerted by age (*b* = 0.001, *t*(212) = 0.21, *p* = 0.83) or gender (*b* = 0.05, *t*(212) = 0.37, *p* = 0.71).

In the second exploratory analysis, we examined whether participants who reported a past diagnosis of ADHD had higher ADHD scores on the ASRS and higher IIV. Two *t* tests were conducted to test these hypotheses on the 215 (out of 218) participants who chose to respond to the previous diagnostic question. The result of the first *t* test indicated that individuals who reported a past diagnosis of ADHD did, indeed, obtain higher ADHD scores: 0.73 *v.* −0.35, *t*(213) = 8.41, *p* < 0.001. The second *t* test revealed that participants who reported a past diagnosis of ADHD presented higher IIV: 0.24 *v.* −0.11, *t*(213) = 2.40, *p* = 0.017.

It is important to note that, while this finding supports our hypothesis, the results reported in the previous (pre-registered) section offer the advantage of measuring ADHD symptoms continuously, rather than relying on a formal, binary diagnosis. As illustrated in Fig. 1, the relationship between ADHD symptoms and IIV spans the entire scale, indicating that the association exists across the full range of ADHD symptom levels, rather than solely among participants with high ADHD symptoms.

In the third exploratory analysis, we sought to investigate the role of the two main components of ADHD symptoms, namely inattention and impulsivity. For each participant, we computed an inattention score based on the nine ASRS items that assess inattention, and an impulsivity score based on the remaining nine ASRS items, assessing impulsivity–hyperactivity. The correlation between the two scores was very high (*r* = 0.89, *t*(216) = 28.92, *p* < 0.001). This extremely high multi-collinearity between inattention and impulsivity statistically prevented us from testing the relative influence of these two constructs.

Finally, the results of all aforementioned hypotheses with linear mixed-effect models are provided in the online Supplementary Materials reported and offer the same conclusions.

### Discussion

Study 1 tested the association between ADHD symptoms and IIV in a numeracy judgment task. The results indicated that participants with stronger ADHD symptoms also exhibited higher IIV. Importantly, this relationship remained significant even after controlling for participants' accuracy. Similar patterns emerged when controlling for age and gender, as well as when comparing the IIV of participants who reported a previous ADHD diagnosis to that of those who did not. Taken together, these findings strengthen the conclusion that stronger ADHD symptoms are linked to increased IIV in numeracy judgment tasks.

While the aforementioned findings support our hypothesis, Study 1 has certain limitations. First, participants made estimations under low-stakes conditions. The only extrinsic motivation was the expectation of gaining an extra $1 in payment for each of the two sets of estimations. Beyond this motivation, participants' performance had no real consequences for them. Related to this point, as altered sensitivity to reinforcement was reported for participants with ADHD (Luman, Tripp, & Scheres, 2010), the inclusion of a payment might have a differential effect on participants with stronger symptoms. Second, ADHD scores (as well as previous ADHD diagnosis) were based on self-reports. Third, IIV was estimated based on relatively few stimuli (maximum of nine jars). Fourth, the study was based on a relatively small number of participants (*N* = 218). Fifth, Study 1 tested our hypothesis with a simple numeracy task, namely, estimating the number of candies in jars. To address these limitations and extend the generalizability of the findings to verbal and quantitative reasoning, we conducted Study 2.

### Study 2: verbal and quantitative reasoning

We addressed the limitations of Study 1 in several ways. First, we collected data from real examinees who had taken a high-stakes admissions test to higher education (here, no monetary reinforcement was involved). Second, the existence of ADHD was determined on the basis of an official diagnosis, previously made by a licensed professional, communicated in documentation provided to the admissions-test administrator verifying adherence to the DSM-5 criteria for ADHD. Third, IIV was assessed using dozens of items. Fourth, the sample-size limitation was addressed by collecting data from more than 3000 examinees with a formal ADHD diagnosis and a control group of more than 200 000 examinees. Finally, the admissions test in question assesses performance in our two domains of interest: verbal and quantitative reasoning. In addition to these abilities, it also tests English as a Foreign Language; therefore, we also analyzed whether examinees with ADHD exhibited higher IIV in this domain.

### Method

#### Data collection

The data were collected under the auspices of the National Institute for Testing and Evaluation (NITE). NITE is mandated by Israeli public universities in Israel and is in charge of the Psychometric Entrance Test (PET), a mandatory selection test for higher education in Israel. NITE is tasked with PET-related research and the design, administration, and score-reporting for the test itself, as well as the collation of relevant documentation from examinees, such as diagnostic reports supporting requests for accommodations. Thus, it was in a position to provide us with all the relevant data.

Our dataset consisted of more than 200 000 examinees who had taken the PET during the period 2012–2020. The study used only the scores obtained from examinees who were being tested for the first time, in order to control for the impact of re-testing.

#### Materials

The PET is a high-stakes standardized test administered in a pencil-and-paper format. It comprises approximately 130 multiple-choice questions and lasts about three hours. The test assesses cognitive abilities in verbal reasoning and quantitative reasoning, together with proficiency in English as a Foreign Language. Each of the three domains is assessed by means of two different but equivalent chapters, each having a similar structure. The quantitative reasoning domain consists of quantitative problems and graphical inference (20 items in total in each chapter). The verbal reasoning domain consists of analogies, inference items, and reading comprehension (23 items in total in each chapter). The English Language domain consists of sentence completions, analogous restatements, and reading comprehension (22 items in total in each chapter). The online Supplementary Materials display several examples of items used in the PET.

#### ADHD diagnosis

The present study examined the data of 3243 examinees with a diagnosis of ADHD who had applied for, and received, test accommodations when sitting the PET. The ADHD diagnosis was confirmed (by NITE's experts at the point at which examinees had applied for accommodations) on the basis of a previously obtained diagnosis made by a licensed professional, in accordance with the Israeli Ministry of Health's current guidelines for assessing ADHD in adults. The assessment involved adherence to DSM-5 (APA, 2013) criteria, administrating ADHD rating scales and supplemental tests, conducting a physical examination, and excluding other potential (differential) diagnoses.

To be eligible for test accommodations, individuals had had to meet strict criteria and provide extensive documentation confirming the following: (a) the onset of ADHD symptoms before the age of 12; and (b) significant impairment in academic, social, occupational, or other areas of performance due to these symptoms. Examinees who received accommodations were allowed a five-minute break after every two chapters. Some of them were also provided with an enlarged answer sheet and basic calculators.

We divided the sample into two groups. The first group consisted of 703 examinees who were not granted accommodations on their initial PET test, but eventually received them on a subsequent attempt at the test (e.g. as they did not provide all required documentation for ADHD, such as previous diagnosis, before the first test). We will refer to this group as the ADHD-no accommodation group. We will compare the IIV (see below) of examinees in this ADHD-no accommodation group to the examinees in a control group of 240 995 examinees who did not apply for and did not receive accommodations. Examinees in the ADHD-no accommodation group provide us with a natural experimental group of individuals with ADHD who took a test identical to that of examinees in the control group, under identical conditions. As specified earlier, we used only the scores obtained from examinees who were being tested for the first time, in order to control for the impact of re-testing.

The second group consisted of 2540 examinees who did receive accommodations for ADHD. We will refer to this group as the ADHD-accommodation group. We will compare the IIV (see below) of examinees in the ADHD-accommodation group to the IIV of a control group consisting of 214 342 examinees who did not apply for and did not receive any accommodation on their tests. Here, too, we used only the scores obtained from examinees who were being tested for the first time.

Note that the number of examinees in the control and ADHD groups differs in the two comparisons because we include, in each comparison, only those examinees from the control group who sat the PET on similar dates to the examinees in the ADHD groups, in order to control for possible differences between test versions.

### Results

The score for each examinee was calculated by computing the percentage of correct answers. Scores were computed separately for each of the two chapters comprising the three domains (quantitative reasoning, verbal reasoning, and English Language proficiency). The primary dependent variable, IIV, was defined as the absolute difference between the scores for the first and second chapters of the same domain. For example, to calculate the IIV for a specific examinee in the quantitative reasoning domain, we determined the absolute difference between the scores for the first and second chapters assessing quantitative reasoning. To control for cognitive ability, we computed for each examinee the average score across the two chapters. Table 1 presents the mean of the IIV, for the ADHD-no accommodation *v.* control groups, by domain.

**Table 1.** Mean of IIV for ADHD-no accommodation and control groups, by domain

Group	N	Quantitative	Verbal	English
Control	240 995	11.03	10.16	9.36
ADHD-no accommodation	703	12.57	10.62	10.18

We analyzed the data using mixed-effect models. In the first model, the dependent variable was the IIV. The fixed effect was the group (coded as 0 for Control and 1 for ADHD-no accommodation). The random effect was one intercept by examinee. The results revealed that, on average, IIV was higher in the ADHD-no accommodation group compared to the control group (*b* = 0.38, *p* < 0.001). As shown in Table 1, higher IIVs were obtained for the ADHD group in all three domains. It is worth noting, however, that although the results were statistically significant, the effect sizes were small. Based on simple averages and standard deviations, we estimated Cohen's *d* to be: 0.17, 0.06, and 0.10, for the quantitative, verbal, and English Language domains, respectively.

In the second model, the dependent variable was the examinee's score. The fixed effect was the group, and the random effect was one intercept by examinee. The results revealed that, on average, scores were lower among the ADHD-no accommodation group compared to the control group (*b* = −6.03, *p* < 0.001).

The third model explored the relationship between IIV and ADHD when controlling for cognitive abilities (scores). The dependent variable was the IIV. Here, the fixed effects were the examinee's group and the average score. The random effect was one intercept by examinee. The results revealed that, on average, the IIV was higher among the ADHD-no accommodation group compared to the control group (*b* = 0.52, *p* < 0.01). In addition, higher IIV was associated with a lower score (*b* = −0.07, *p* < 0.001).

In the next, we compared the IIV of the examinees in the ADHD-accommodation group to that of the control group. Table 2 presents the mean of the IIV, for the control and ADHD-accommodation groups, by domain.

**Table 2.** Mean of IIV for ADHD-accommodation and control groups, by domain

Group	N	Quantitative		Verbal	English
Control	214 342	11.02		10.14	9.37
ADHD-accommodation	2540	11.34		10.57	9.61

We analyzed the data using mixed-effect models. In the first model, the dependent variable was the IIV. The fixed effect was the group (coded as 0 for Control and 1 for ADHD-accommodation). The random effect was one intercept by examinee. The results revealed that, on average, IIV was higher in the ADHD-accommodation group compared to the control group (*b* = 0.33, *p* < 0.001). As shown in Table 2, higher IIV was obtained for the ADHD group in all three domains. Here, once again, although the results were statistically significant, the effect sizes were small. Based on simple averages and standard deviations, we estimated Cohen's *d* to be 0.09, 0.07, and 0.07, for the quantitative, verbal, and English Language domains, respectively.

In the second model, the dependent variable was the examinee's score. The fixed effect was the examinee's group and the random effect was one intercept by examinee. The results revealed that, on average, scores were lower among the ADHD-accommodation group compared to the control group (*b* = −1.13, *p* < 0.001).

The third model explored the relationship between IIV and ADHD when controlling for cognitive abilities (scores). The dependent variable was the IIV. Here, the fixed effects were the group and the average score. The random effect was one intercept by examinee. The results revealed that, on average, the IIV was higher among the ADHD-accommodation group compared to the control group (*b* = 0.25, *p* < 0.01). In addition, higher IIV was associated with a lower score (*b* = −0.07, *p* < 0.001).

### Discussion

Using a real-life, high-stakes situation, Study 2 explored the IIV of the scores of examinees who had taken the PET for the first time. We conducted two analyses. In the first analysis, we compared the IIV of examinees with ADHD who did not receive accommodations in the test to that of examinees without ADHD. Higher IIV was observed in the ADHD group. This finding offers a comparison between examinees with and without a formal ADHD diagnosis, taking the same test under identical conditions, and thus free from confounding effects and the effect of irrelevant variables.

In the second analysis, we compared the IIV of examinees with ADHD who received accommodations to that of a control group. Here, too, higher IIV was observed in the ADHD group. This finding further reinforces the results of the first analysis by demonstrating that higher IIV was observed among examinees with ADHD, even after they received test accommodations. Even though it appears that effect sizes were lower in the ADHD-accommodation condition compared to the ADHD-no accommodation condition, this analysis points to the pervasive effect of ADHD, illustrating how it continues to impact examinees even after considerable efforts to mitigate its effects.

One possible explanation for the small effect sizes is that examinees with ADHD probably took medications that, in addition to helping them remain focused during the test, also reduced their IIV. Indeed, the literature reports that increases in reaction-time variability among individuals with ADHD can be attenuated, and even normalized, with stimulant medication (Tamm et al., 2012).

## General discussion

The goal of the present study was to investigate fluctuations in behavioral and cognitive performance among individuals with ADHD, in two previously unexplored cognitive domains: numerosity judgments and verbal and quantitative reasoning. These fluctuations were captured by the IIV measure.

In Study 1, the participants’ task was to estimate the number of candies in jars, on two separate occasions. IIV was defined as the absolute distance between the estimates made on the two occasions for the same stimuli. We found that participants with stronger ADHD symptoms exhibited higher IIV compared to those with weaker ADHD symptoms. Importantly, this result held even after controlling for the participants’ accuracy.

In Study 2, we investigated IIV in performance on complex cognitive tasks. We collected data on more than 200 000 examinees who had taken a high-stakes admissions test for selection to higher education, of whom more than 3000 had a formal diagnosis of ADHD. IIV was defined as the absolute distance between the scores for two exam chapters assessing the same cognitive abilities. We found that individuals with ADHD (both with and without accommodations) exhibited higher IIV compared to the control group. Here, too, the results remained consistent even after controlling for the examinees' scores.

### Theoretical implications

Investigating IIV in performance on complex cognitive tasks such as verbal and quantitative reasoning could provide valuable insights into the cognitive processes that characterize individuals with ADHD. In addition, it has the potential to help improve the accuracy of cognitive and academic assessment and measurement in this population. To the extent that individuals with ADHD do exhibit increased IIV, it would suggest that the measurement of the same psychological constructs is less precise in this population. This may also imply that the same constructs should be assessed differently for people with and without ADHD. In this respect, it is worth noting that Study 2 analyzed data obtained in a naturalistic setting: from real examinees who were highly motivated to maximize their potential in order to increase their chances of being accepted into universities.

Our study may hold significant implications for the ADHD literature. Until now, the scholarship has established the connection between ADHD and IIV mainly in terms of processing speed (mean reaction time). This study, however, establishes the connection with regard to the quality of performance in judgment and complex cognitive tasks. This finding underscores the pervasive nature of IIV as a characteristic of ADHD, signifying that individuals with this disorder not only exhibit inconsistency in reaction speed to stimuli over time but also demonstrate less consistent numerosity judgment and greater fluctuation in higher cognitive functioning.

Assuming that ADHD relates to increased IIV in other judgment domains, it may be concluded that ADHD-related judgments are characterized by increased ‘noise’ (variance) in addition to decreased accuracy. Judgment and other cognitive abilities are closely related to decision-making. Thus, when an individual evaluates options and judges one of them to be favorable, they are more likely to choose that option. Having a more fluctuating distribution of judgments means that, over time, there is a higher likelihood of making variable and even contradictory choices. Indeed, secondary analyses of decision-making tasks suggest that the assessment of risk performed by individuals with ADHD presents less consistency across trials compared to individuals without ADHD (Pollak et al., 2020). Similarly, ADHD has been associated with less consistent intertemporal choices involving a smaller but more immediate reward and a larger but more delayed reward (Gabrieli-Seri et al., 2022).

Higher IIV in the evaluation of important decision-making parameters may lead to extreme choices toward both ends of the caution–incaution spectrum. For instance, consider choices involving risk: variability in risk judgment increases the probability of engaging in excessively risky behavior, such as taking dangerous actions that others would avoid. This aligns with the association between ADHD and unwarranted risk-taking behavior (Pollak, Dekkers, Shoham, & Huizenga, 2019). Conversely, higher IIV in risk judgment also elevates the probability of engaging in excessively cautious behavior, meaning one might avoid risks that others would deem acceptable. Indeed, this conclusion aligns with findings from several studies and meta-analyses (Dekkers et al., 2021; Dekkers et al., 2020; Golm et al., 2020; Roberts, Alderson, Betancourt, & Bullard, 2021).

### Practical implications

The increased IIV in ADHD also holds clinical implications. First, as IIV is characteristic of ADHD, adding an assessment for performance consistency may contribute to the characterization of an ADHD patient. The connection between ADHD and more inconsistent judgment as well as higher fluctuation in cognitive abilities implies that interventions for individuals with ADHD should target the inconsistency of their performance and decision-making processes. Increased IIV in performance might lead to frustration and confusion for the individual about their self-efficacy, which practitioners need to take into account. In addition, when supporting clients with ADHD, it is essential to work on helping them avoid making extreme judgments, be it by refraining from inaccurately perceiving very risky options as not so risky, or by learning to correctly judge less risky or low-risk options for what they are.

Finally, the increased IIV in performance on complex cognitive abilities impacts the accuracy of the assessment and measurement of various variables among individuals with ADHD. It suggests that the measurement of the same psychological constructs is less precise among those with ADHD. Consider an admissions test with a specific cutoff score, in which individuals who score beyond the cutoff are accepted, whereas those who score below it are not. The likelihood that an examinee whose actual ability is above the cutoff will score *below* it on a given occasion is higher among individuals with ADHD than among examinees without ADHD who have the same level of ability. Notably, the likelihood that an examinee whose actual ability is below the cutoff will score *above* it is also higher among individuals with ADHD than among examinees without ADHD who have the same level of ability. To mitigate the impact of this variability, aggregating the results of multiple assessments becomes particularly important to overcome such ‘noise’. Given the higher level of variability in the performance of individuals with ADHD, including more assessments is necessary to obtain more accurate estimates.

## Supporting information

Barneron et al. supplementary material Barneron et al. supplementary material

## Supplementary material

The supplementary material for this article can be found at https://doi.org/10.1017/S0033291724001892.
