# Supplementary material for: Increased intra-individual variability among individuals with ADHD: first evidence from numerosity judgment and verbal and quantitative reasoning
Source: Psychol Med. 2024 Oct 23;54(13):3689–96. doi: 10.1017/S0033291724001892 (PMC11536105; doi:10.1017/S0033291724001892)
Supplement: Barneron et al. supplementary material [file S0033291724001892sup001.docx]

**Supplementary Materials**

These Supplementary Materials are divided into two parts. The first part sets out the additional analyses conducted in Study 1 (mixed-effect models), as described in the Results section of the article. The second part shows examples of the items used to assess performance in the three domains of the Psychometric Entrance Test.

**Study 1. Numerosity Judgments: Additional Analyses**

In the mixed-effect models, IIV was computed as the absolute distance between the estimates in Phase 1 and 2 for each participant and jar, using raw (i.e., non-standardized) measures. Absolute Error (hereafter, AE) was defined as the absolute difference between the estimates and the actual number of candies in Phases 1 and 2.

The mean AE was then calculated for each participant and jar, by averaging the AE across Phases 1 and 2. This captures participants’ performance accuracy across both phases for each participant and jar. The ADHD score was defined at the participant level and computed as the average of the 18 items on the ADHD questionnaire.

The first model tested the association between ADHD and IIV. In the first mixed-effect model, the dependent variable was the IIV. The fixed effect was ADHD, and the random effects were one intercept by participant and one intercept by jar. The results demonstrated a positive association between ADHD scores and IIVs (*b* = 11.97, *p* = .001).

The second model tested the association between ADHD symptoms and the AEs. Here, the dependent variable was the AE. The fixed effect was ADHD, and the random effects were one intercept by participant and one intercept by jar. The results did not show a positive association between ADHD scores and AEs (*b* = 2.24, *p* = .38).

In the third model, we investigated the relationship between IIV and ADHD while controlling for participants’ performance accuracy. Here, the dependent variable was IIV. The fixed effects were the ADHD scores and the AEs. The random effects were one intercept by participant and one intercept by jar. The results indicated that ADHD scores were positively associated with IIVs (*b* = 11.28, *p* < .001). In addition, the AEs were also positively associated with IIVs (*b* = 0.32, *p* < .001). Therefore, ADHD scores can be said to be positively associated with IIV, even after controlling for participants’ performance accuracy.

The fourth model was similar to the third model but, here, the random effects were one intercept by participant, and one intercept and coefficient of ADHD scores by jar (the model failed to converge when attempting to add the AEs as random effects by jar). The results of this model indicated that ADHD scores remained positively associated with IIV (b = 11.23, *p* = .001). Here, too, the AEs were positively associated with IIVs (*b* = 0.31, *p* < .001).

## Exploratory Analyses

The exploratory analyses reported in the main manuscript were analyzed with the following mixed-effect models.

The influences of age and gender were tested with a model built similarly to the fourth model. The dependent variable was IIV. The fixed effects were the ADHD scores, the AEs, age, and gender. The random effects were one intercept by participant and one intercept by jar. The results indicated that ADHD scores were positively associated with IIVs (*b* = 11.48, *p* < .001). In addition, the AEs were also positively associated with IIVs (*b* = 0.32, *p* < .001). We found no effect of age (*b* = 0.08, *p* = .71) or gender (*b* = 1.30, *p* = .78).

The difference in IIV between those participants who reported having a previous ADHD diagnosis and those who did not was assessed with a mixed-effect model where the dependent variable was IIV. The fixed effect was the participant’s group (diagnosed vs. not diagnosed; the model predicted the diagnosed group). The random effects included one intercept by participant and one intercept by jar. (A model including also a random coefficient for the diagnosed group, by jar, did not converge.) The results indicated that participants who reported having a previous ADHD diagnosis had higher IIV compared to those who did not: *b* = 13.03, *p* = 015.

**Study 2. Verbal and Quantitative Reasoning: Examples of Items Used in the Israeli Higher Education Psychometric Entrance Test**

The Psychometric Entrance Test (PET) consists of nine sections, each of which belongs to one of the following domains: Verbal Reasoning, Quantitative Reasoning, or English Language Proficiency.

The first section of the test is part of the Verbal Reasoning domain and consists of a writing task. Those examinees choosing to take the test in Hebrew have 30 minutes to complete this section (examinees who choose to take the test in other languages are given 35 minutes). The one task that is covered by this section was not included in our study as examinees are asked to write only one (and not two) essays so it was not possible to investigate IIV with respect to writing abilities.

The remaining eight sections are made up of multiple-choice questions for which the response that best answers the question must be chosen from among four alternatives. These sections do not appear in any particular order, and all examinees have 20 minutes in which to complete them.

The multiple-choice sections in each domain consist of several types of questions. All questions of a given type appear together and are arranged in ascending order of difficulty, except for Reading Comprehension questions (in the Verbal Reasoning and English domains), which are arranged according to the order in which the subject matter appears in the text.

Of the eight multiple-choice sections, only two in each domain (a total of six) are used for calculating the score. The multiple-choice sections that are not used for calculating the score serve two purposes: to prevent differences between tests and to ensure the quality of the questions.

## Verbal Reasoning

### **Writing Task**

The writing task consists of producing an essay on a given topic. The score on the writing task constitutes 25% of the Verbal Reasoning score. The following instructions appear at the beginning of the section:

This section consists of a writing task. The time allotted is 35 minutes. Read the task carefully and write your essay on the lined answer sheet provided. The essay must be at least 25 lines long. Do not write an essay that is longer than the number of lines on the answer sheet. If you need scrap paper, use pages 2 and 3 of the test booklet. (This draft will not be marked.) You will not receive an additional answer sheet, nor will you be allowed to replace the one you have.

Use a style that is consistent with academic writing. Make sure your essay is well organized and written in clear, grammatically correct language.

*Example of Writing Task*

In the past, the voting age in general elections in most countries was twenty-one, but in the last several decades there has been a worldwide trend to reduce the voting age to eighteen. Some countries, including Israel, have introduced bills to reduce the voting age from eighteen to seventeen, and even to sixteen, and several countries have already done so. The public discourse on reducing the voting age centers on questions such as: At what age should a person be entitled to choose the government officials whose decisions have a direct bearing on his life? Are teenagers mature enough to make decisions that affect the entire population? What are the implications of including teenagers in the voting population and involving them in the affairs of the state and in the political system in general?

In your opinion, should the voting age be reduced? Give reasons. You may make use of the questions posed in the text.

### **Analogies**

Each of the following questions contains a pair of words in bold type. Find the relationship between the meanings of these two words. Then choose from among the possible responses the one in which the relationship between the two words is most similar to the relationship you have found.

Note: The order of the words in each pair is significant.

**baker : eating**

1. surgeon : anesthesia
2. author : reading
3. gardener : planting
4. policeman : enforcement

**Critical Reading and Inference Questions**

This section examines your ability to read and understand complex material and draw valid conclusions from it. You will be asked to understand a statement’s internal logic, to understand and apply principles of logic, and to compare different ideas and situations. In addition, you will be required to understand texts taken from a wide range of sources and encompassing a wide variety of writing styles: articles, textbooks and scientific journals, as well as newspapers and other sources.

Most of the critical reading and inference questions contain information or a short text and a question that relates to them. Sometimes, several questions may be based on the same information and the same instructions.

#### *Example 1*

Charles Leadbeater: “The personal computer I am using to write this essay is composed of approximately the same amount of plastic, gold, silicon, copper, and other metals as the computer I used five years ago. Both pieces of equipment are fairly similar in weight and shape, but my present computer is twenty times more powerful than my previous computer. This difference is due to human intelligence, which rearranges the available materials in order to obtain more from them. This is the story of the economic growth that has occurred in the modern era.”

According to Leadbeater, what is “the story of the economic growth that has occurred in the modern era”?

1. Man’s wiser use of available resources makes it possible to obtain more from them.
2. The constant development of new technologies makes it possible to produce a greater variety of materials.
3. The personal computer which man developed makes it possible to do calculations faster and more powerfully than ever before.
4. The improvement in human intelligence in the modern era has led to accelerated economic growth.

#### *Example 2*

This year ______ tourists visited Rome than last year; however, ________to be the biggest tourist destination in Italy. Travel agents explain that the number of tourists to Italy has _______ relative to previous years, _______ have chosen to visit Rome.

1. more / for the first time in many years it does not appear / increased / and it seems that most
2. fewer / as in the past, it appears / increased / and it seems that only a small proportion
3. fewer / for the first time in several years it appears / in fact decreased / but it seems that most
4. more / this year as well, it appears / in fact decreased / but it seems that a large proportion

#### *Example 3*

After Shulie and her friend watched a televised announcement by one of the government ministers, Shulie said: “It is as if he is saying, ‘I’ll break the barrel and keep its wine.’”

Which of the following is most likely to be the announcement to which Shulie was referring?

1. Minister of Education: “The education budget will be cut, but the level of education will not be affected.”
2. Minister of Transport: “I will speed up the laying of the railway track despite technical problems.”
3. Minister of the Treasury: “Taxes will not be reduced next year, despite the government’s promise.”
4. Minister of Defense: “In spite of what military commentators say, the professionalism of army officers has actually increased.”

### **Text Comprehension Questions**

Instructions: Read the text below carefully and answer the questions that follow.

#### *Example of Text Comprehension*

For almost three hundred years man has been using animals for research in order to learn from the animals’ cerebral, physiological, and behavioral mechanisms to better understand corresponding mechanisms in humans. Almost from the start, controversy arose as to whether using animals in this way was morally justified.

Until the 18th century in Europe, all aspects of life, including science, were governed by a Christian religious perspective. According to this view, God created mankind in His image, and He created all other creatures to serve man. Thus, man is permitted to use animals for his own needs. Secular philosophers, as well, maintained that man has no moral obligation towards animals: animals do not have the ability to use language; they therefore do not have beliefs, ambitions, or desires, and thus do not have interests that must be protected.

Objections to harming animals were voiced for the first time at the end of the 18th century. English philosopher Jeremy Bentham asserted that the question that should be asked in this regard is not whether or not animals have awareness but, rather, whether they are able to feel pain, to which the answer is “yes.” Bentham’s successors also disagreed with the view that animals do not have beliefs and desires. They argued that a dog can believe that a certain bone is tasty even if it is incapable of formulating a sentence to that effect.

The controversy became more acute during the second half of the 19th century with the introduction of Charles Darwin’s theory of evolution. Darwin maintained that animals and man have a common origin, and pointed to the physiological similarities among the different species. This further reinforced the belief that findings from experiments on animals could be applied to humans. However, since the theory of evolution placed man and animals on a single, continuous developmental axis, it was difficult to argue that only humans were capable of suffering or feeling pain.

A compromise was proposed in the 1970s by Australian philosopher Peter Singer. Singer suggested that the principle of benefit versus harm be applied whenever an experiment on animals was being considered. According to this principle, the amount of good to be derived from the experiment – for humans and for animals – should be weighed against the amount of suffering it would cause, and the experiment should be conducted only if benefits outweighed harms. Singer, however, asserted that the interests of humans and those of animals do not carry equal weight. Thus, for example, in the case of a sinking ship, it is better to sacrifice the life of a dog than that of a human being. Singer’s opponents argued that nature is governed by the principle of survival of the fittest, and therefore, any use that humans make of animals for their own needs – and certainly any designed to improve their chances of surviving – is justified.

In recent decades, advocates of the restriction and even of the total prohibition of the use of animals for research purposes are becoming increasingly vocal. The scientific community has formulated several guiding principles in this regard: to perform experiments on animals only if they are likely to bring real benefit to the human race; to make every effort to minimize the pain and suffering caused to animals during the course of an experiment; whenever possible, to give preference to alternative methods of research (such as computer imaging); and so on. Medical schools are attempting to instill these values in their students. For example, in one course on research methods, students were required to plan an animal experiment to test the efficacy of a medication, and then were required to find a way of testing its efficacy by means of research that did not involve animals.

*Questions:*

1. It can be inferred from the second paragraph that “secular philosophers” (line 8), ___________individuals with a Christian religious perspective, maintained that using animals for the needs of man was justified and that proponents of each approach______________.

1. as well as / justified their position using a different argument
2. as opposed to / presented moral arguments to support their position
3. as well as / objected only to the use of animals for research purposes
4. as well as / explained themselves on the grounds that animals do not have the ability to use language

2. The view referred to in line 15 is:

1. that animals have awareness
2. that harming animals is immoral
3. that of the individuals with the religious perspective mentioned in the second paragraph
4. that of the secular philosophers mentioned in the second paragraph

## Quantitative Reasoning

### **Questions and Problems**

#### *Example 1*

A driver traveled from Haifa to Eilat. He covered a third of the distance at a speed of 75 kilometers per hour (kph), a fifth of the remaining distance in one hour, and the rest of the distance at a speed of 80 kph. The distance between Haifa and Eilat is 450 kilometers. If the driver had driven the entire distance at a constant speed, at what speed would he have needed to drive so that the journey from Haifa to Eilat would take exactly the same amount of time?

1. 70 kph
2. 75 kph
3. 80 kph
4. 90 kph

#### *Example 2*


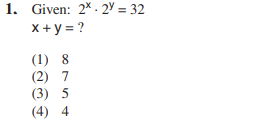


#### *Example 3*


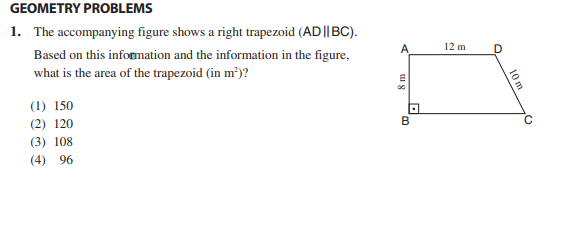


### **Graph and Table Comprehension**

#### *Example 1
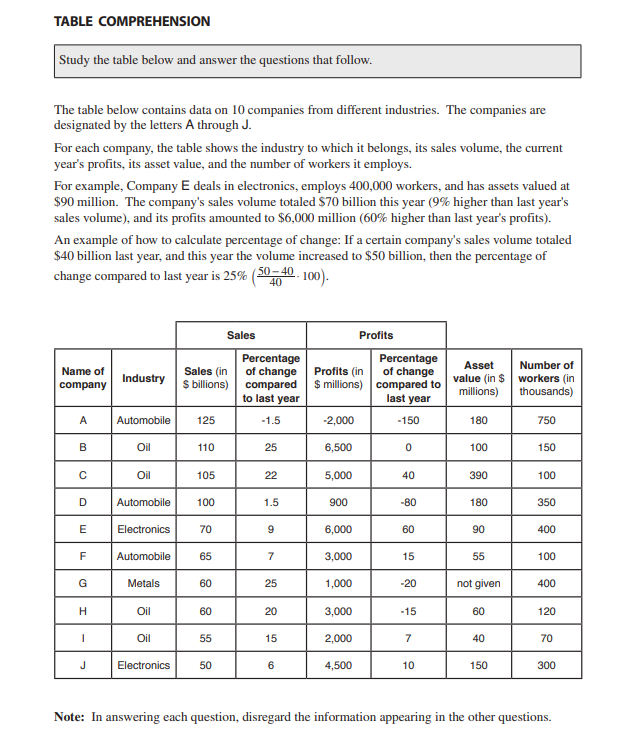
*

Which of the companies in the automobile industry has/have the lowest asset value?

1. A
2. D
3. F
4. A and D

#### *Example 2*


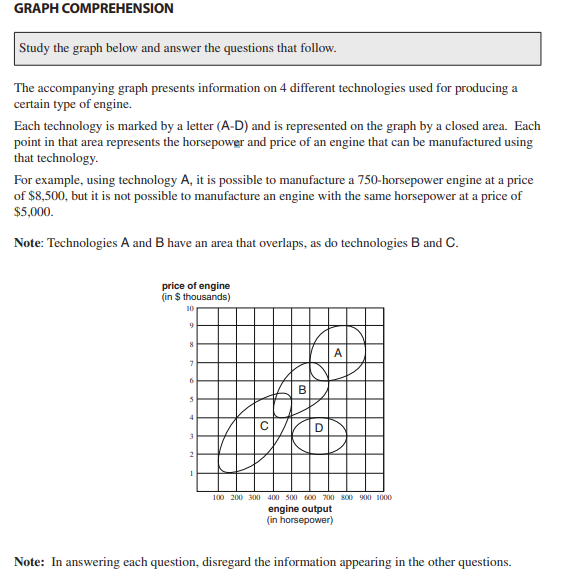


What is the range of engine outputs (in horsepower) that can be obtained using technology A as well as technology B?

1. 400–500
2. 500–600
3. 600–700
4. None of the above

## English

### **Instructions:**

### The following section contains three types of questions: Sentence Completion, Restatement, and Reading Comprehension. Each question is followed by four possible responses. Choose the response that best answers the question and mark its number in the appropriate place on the answer sheet.

### Sentence Completion

### This part consists of sentences with a word or words missing in each. For each question, choose the answer that best completes the sentence.

“Most psychologists today believe that adopted children should be permitted and even ___________ to learn about their biological parents.”

1. encouraged
2. endured
3. enriched
4. enclosed

### Restatements

### This part consists of several sentences, each followed by four possible ways of restating the main idea of that sentence in different words. For each question, choose the one restatement that best expresses the meaning of the original sentence.

“Analysts claim that an increase in exports is responsible for Poland’s economic recovery.”

1. Analysts suggest that growth in Poland’s economy can be achieved by increasing exports.
2. Analysts believe that only if Poland’s economy recovers will the country be able to export large amounts of goods.
3. According to analysts, the improvement in Poland’s economy is the result of increased exports.
4. According to analysts, the recent growth in Poland’s economy will encourage the export of more goods.

### Reading Comprehension

This part consists of two passages, each followed by several related questions. For each question, choose the most appropriate answer based on the text:

One afternoon in 1993, an American gem expert named Benjamin Zucker received an unexpected visit from a Swiss gem dealer. The dealer placed a plain box on Zucker's desk and casually opened it, revealing 23 pearls, so large and of such a brilliant orange color that Zucker thought they could not possibly be genuine. The largest was 32 millimeters in diameter – bigger than a robin’s egg. Zucker had seen few pearls of that size and had never seen an orange pearl of any kind. The Swiss dealer knew nothing of the pearls’ history, except that they had been purchased in Vietnam and were said to have come from the imperial treasury. Though the pearls were not for sale, Zucker became determined to trace their origins.

Zucker took the pearls to Kenneth Scarratt at the Gemological Institute of America. Scarratt confirmed that the pearls were real and that they were almost definitely from Vietnam. Scarratt had seen one such pearl several years before and had traced it to a type of shell found in Vietnamese waters. He reported that only four orange pearls had ever been documented, all in the past 30 years and all from Vietnam. He was astounded to see such a large collection. Scarratt’s information suggested to Zucker that, indeed, the pearls must have belonged to Vietnamese royalty: in a country like Vietnam, where wealth and power had been concentrated for generations in the hands of the emperors, no one else could have acquired such an extraordinary collection. Scarratt also told Zucker that the pearls were probably hundreds of years old. He based his conclusion on a pattern of tiny wear marks, which indicated frequent handling over a long period of time.

Zucker set out to learn everything he could about pearls in Vietnamese art and history. He found that many 18th- and 19th-century Vietnamese royal objects featured a pearl with a flaming tail, often pursued by a dragon, the symbol of the Vietnamese emperor. This suggested to Zucker that the Vietnamese emperors had indeed owned flame-colored – that is, orange – pearls. Zucker then traveled to Vietnam, where he met with scholars and with people who had been close to the royal family. None of them had seen or even heard of such pearls. Nor could he find any record of the collection. Nonetheless, Zucker remains convinced that the pearls were once the treasure of the emperors of Vietnam.

*Questions:*

1. According to the first paragraph, when Zucker first saw the pearls, he thought that they were:

1. Vietnamese
2. not for sale
3. stolen
4. not real
